# Discriminating Global Orientation of Two Element Sets

**DOI:** 10.5334/joc.127

**Published:** 2020-10-13

**Authors:** Aytaç Karabay, Daniel D. Kurylo

**Affiliations:** 1 Department of Psychology, Experimental Psychology, University of Groningen, Groningen, NL; 2 Department of Psychology, Brooklyn College CUNY, Brooklyn, NY, US

**Keywords:** grouping and segmentation, spatial integration, global orientation, perceptual grouping

## Abstract

Perceived global organization of visual patterns is based upon the aggregate contribution of constituent components. Patterns constructed from multiple sources cooperate or compete for global organization. An investigation was made here of interactions between two interspersed element sets on global orientation. It was hypothesized that each set would operate as an integrated unit, and contribute independently to global orientation. Participants viewed a 10 × 10 array of Gabor patches, and indicated the predominant orientation of the array. In Experiment 1 all elements were rotated. Rotation up to 23° had little effect, whereas greater rotation produced a progressive shift on global orientation. In Experiment 2 a proportion of elements remained aligned while remaining elements were rotated. Embedding a proportion of aligned elements stabilized global orientation, which was dependent upon the proportion of aligned elements. Specifically, with 20% alignment, global orientation was similar to rotating all elements, whereas 80% alignment strongly biased perception towards aligned elements. The stabilizing effect varied with rotation of the second element set. Across levels of rotation, alignment effects rose to a peak then declined as element sets became orthogonal. In Experiment 3, each element set was rotated independently. Independent rotation of both sets altered global orientation, compressing the psychometric function for the single-element condition. Together, for interspersed element sets with explicit orientations, each set does not contribute independently to global orientation. Instead, element sets interact, where the contribution of one set, presented at a fixed rotation and fixed proportion, varies with the change to the second set.

Global perception of a field of individual elements, whether constructed from a uniform element set, an element set that varies across a defined distribution, or multiple sets with discrete properties, entails the integration of stimulus components. In each case, global perception is based upon the aggregate contribution of elements, where stimulus components collectively produce a coherent structure (Kimchi, 1998; Kimchi, Hadad, Behrmann, & Palmer, 2005). Examining the formation of global patterns, local elements are organized into global configurations early in processing, where factors such as collinearity of line segments contribute to stimulus organization (Kimchi, 2000).

Perception of global structure appears to represent a distinct process. Evidence supporting a distinct process for global structure include proposed neural correlates, where specific regions within visual cortex are associated with global perception, including contours formed by collinear line segments (Kourtzi, Tolias, Altmann, Augath, & Logothetis, 2003) and perception of hierarchical patterns (Huberle & Karnath, 2012; Ritzinger, Huberle, & Karnath, 2012). In addition, clinical conditions exist in which local components or forms are perceived normally, while perception of global patterns or multiple objects is impaired (Behrmann & Kimchi, 2003; Huberle & Karnath, 2010; Huberle, Rupek, Lappe, & Karnath, 2009; Himmelbach, Erb, Klockgether, Moskau, & Karnath, 2009).

Perception of global structure is based upon integration of stimulus components, which is guided by characteristics of elements. Elements that share properties form a unified structure, whereas patterns constructed from elements with conflicting properties form multiple representations that produce competing organization (Rashal, Yeshurun, & Kimchi, 2017). Interactions among stimulus components have been explored with fields of oriented elements that define textures or contours. For orientation-defined textures, an abrupt discontinuity in element orientation produces a boundary between texture fields (Bach, Schmitt, Quenzer, Meigen, & Fahle, 2000; Gray & Regan, 1998; Kwan & Regan, 1998; Popple, 2003; Wolfson & Landy, 1995), whereas textures containing multiple orientation sets within the field produce an integrated structure, or form intermixed element sets (Husk, Huang, & Hess, 2012; Keeble, Kingdom, & Morgan, 1997; Keeble, Kingdom, Moulden, & Morgan, 1995; Parkes, Lund, Angelucci, Solomon, & Morgan, 2001). Processing global patterns with multiple orientation sets has been described as proceeding in two stages: low-level encoding of local properties, followed by integration among neurons tuned to similar features (Achtman, Hess, &Wang, 2003; Dakin & Watt, 1997). Integration may occur by summating local information across space (Jones, Anderson, & Murphy, 2003), or summating an element subset of segregated signals (Husk et al., 2012).

For stimuli containing elements with a coherent orientation that are embedded among randomly oriented elements, increased orientation variance decreased discrimination of global orientation (Husk et al., 2012). Results were modelled as the integration of coherent elements, where elements rotated beyond the sensitivity of orientation filters served as noise, and did not contribute to the global orientation. For stimuli containing two dominant orientations, detecting multiple orientation sets requires a substantial difference in orientation (13°), below which multimodal stimuli cannot be discriminated from stimuli composed of a single orientation set. Results may reflect an integration process that follows an initial stage of orientation filters, or may reflect a grouping mechanism that integrates elements of similar orientation (Keeble et al., 1997).

Orientation coherence serves to enhance integration among constituent elements, which increases the saliency of the aligned elements. Orientation coherence effects are apparent with texture segmentation, in which aligned elements contained within textural regions facilitate texture segmentation. Specifically, orientation-contrast thresholds declined as the level of collinearity increased, suggesting an interaction among orientation detectors (Harrison & Keeble, 2008). Orientation coherence effects are also apparent with contour integration, where collinear, co-axial elements form coherent shapes that are distinguished from randomly-oriented elements (Field, Hayes, & Hess,1993; May & Hess, 2007; May & Hess, 2008). At a local level, alignment modulates interactions among adjacent elements, affecting the detection of targets in the presence of similarly oriented flankers (Polat & Sagi, 1993). Such effects suggest that for stimuli containing mixed orientations, aligned elements disproportionately contribute to global orientation.

Element sets with sufficient difference in orientation appear to form distinct global structures, as reported for orientation-defined texture boundaries, intermixed texture sets, or elements with coherent orientation embedded in randomly oriented elements. Based upon these effects, it was hypothesized that multiple element sets contribute independently to perceived global orientation. To test this hypothesis, an analysis was made here of the interaction between two element sets on global orientation. Stimuli were composed of two element sets, each with an explicit, non-random orientation. Performance with the two-element sets was compared to that of a single-element set, where all elements were rotated similarly. It was predicted that varying the characteristics of one element set will not change the contribution to the global orientation of the alternate set, but instead, each set will operate as an independent structure.

## General Method

### Participants

Undergraduate college students participated in the study in order to receive course credit. All subjects had best-corrected visual acuity of 20/20 (Snellen) at a test distance of 35 cm, and were free from astigmatism (as verified with the Radial Spoke Test). The study was conducted in accordance with the Declaration of Helsinki and approved by the ethical committee of the Psychology Department of the University of Groningen (approval number 15076-NE). Before participating in the study, participants signed an informed consent statement.

### Apparatus and Stimuli

Stimuli were presented on a 22” CRT monitor (iiyama model MA203DT) set to a resolution of 1280 × 1024, 32-bit color depth, and 100 Hz refresh. Stimulus presentation, trial events, and data collection were controlled by E-Prime 2.0 Professional (Schneider, Eschman, & Zuccolotto, 2002).

**Figure 1:**
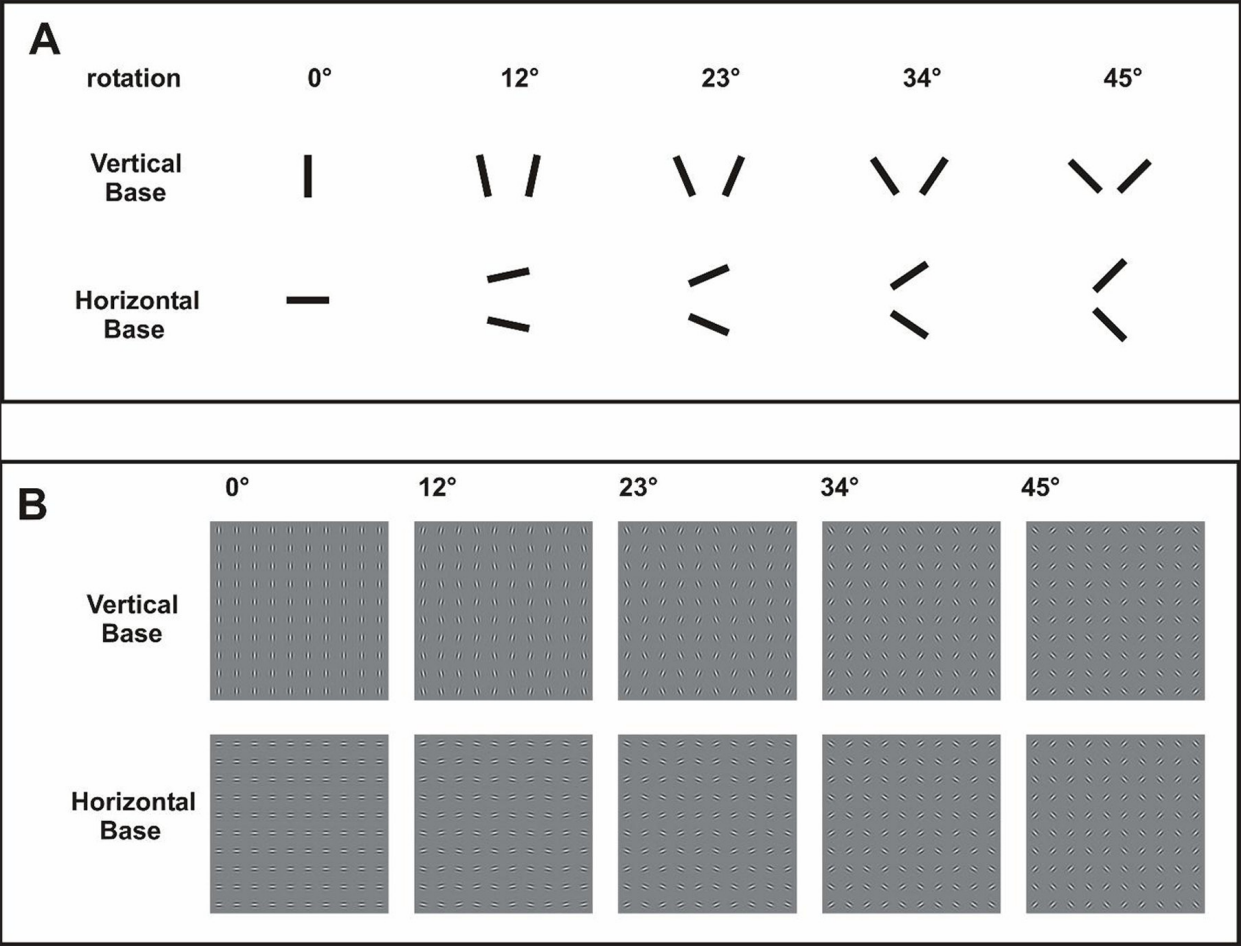
**A**. Elements types for Experiment 1 at each level of rotation. For rotated conditions, one of the two possible orientations (clockwise or counterclockwise, relative to base orientation) was pseudo-randomly selected. **B**. Examples of stimuli at each level of rotation.

Stimuli consisted of a 10 × 10 square grid of Gabor patches on a gray background. Gabor patches were set to 2.44 cpd with an S.D. of 0.2º, in which approximately two cycles were visible. Centers of adjacent patches were separated by 2.0º, and the entire grid subtended 19.6º. The entire array of Gabor patches thereby covered a broad area, including both central and peripheral viewing (Caelli & Moraglia, 1985). Luminance ranged from 7 to 324 cd/m^2^, and background luminance was 73 cd/m^2^. Elements were either aligned to a cardinal orientation (either vertical or horizontal, which randomly varied across trials) (Figure 1), or deviated from the cardinal orientation, either clockwise or counterclockwise, by a fixed level of rotation. Stimulus sets were created a priori, and each stimulus condition contained a horizontal and vertical version. The distribution of element sets was pseudo-randomized and distributed uniformly across the grid, such that the proportion of each set was matched along every column and every row. The algorithm for distributing element set members across the stimulus array included procedures to avoid clumping of element types, which may facilitate texture segmentation. Appendix Table 1 provides a summary of the stimuli parameters used in each experiment.

### Procedure

Participants sat individually in a sound-attenuated testing cabin, and viewed the monitor from a distance of approximately 60 cm. On each trial, either the horizontal or vertical pattern (referred to here as the “base” orientation) was selected randomly. A fixation point initially appeared in the center of the monitor for 250 ms, followed by a 180 ms test stimulus. Following stimulus presentation, subjects indicated whether the stimulus appeared predominantly organized vertically or horizontally (two-alternative, forced-choice). Results are presented as the percentage of trials in which subjects chose the base orientation. For each of the three experiments, data collection was preceded by 25 practice trials that were not used in the analysis.

### Data Availability

Raw data of each experiment are uploaded to Open Science Framework with a unique identifier 2dp6e (https://osf.io/2dp6e/) which is publicly available.

## Experiment 1: Single Element Set

The aim of Experiment 1 was to determine the global orientation of a stimulus array in which all elements were rotated by a fixed amount. These data can then be used as a baseline to examine the effects of two-element stimuli.

### Method

Participants. Five subjects participated in Experiment 1 (mean age 22.4 years; age range 19–29 years; 3 females).

Stimuli. All elements were rotated from the base orientation by either 0º, 12º, 23º, 34º, or 45º. The direction of rotation was either clockwise or counterclockwise element (Figure 1), assigned pseudo-randomly for each element. It should be noted that the number of clockwise and counterclockwise elements were equally distributed across the visual array. Pairs of rotated elements (with clockwise and counterclockwise rotation) thereby bracketed the vertical or horizontal base orientation (Figure 1A). Discriminability of base orientation thereby varied across element rotation, where vertical and horizontal patterns differed greatest with 0º rotation, and patterns became more similar with increased rotation. With 45º rotation, element types for vertical and horizontal patterns were the same, and patterns provided no cue for discrimination. Level of rotation was randomized and interleaved across trials. Performance was based upon 24 trials at each level of rotations. The duration of Experiment 1 was approximately 10 mins per participant.

### Results and Discussion

**Figure 2:**
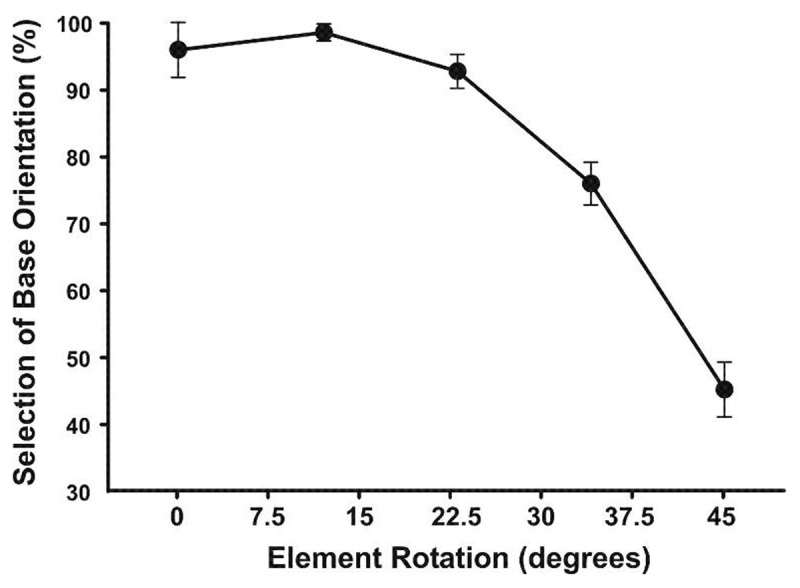
Mean selection of base orientation as a function of element rotation; error bars represent SEM.

As elements deviated from the base orientation, perceived global orientation progressively shifted from the base orientation to chance performance (Figure 2). Analysis of variance (ANOVA), with repeated measures on the rotation factor, indicated a significant effect of rotation (*F*(4,16) = 50.84, *p* < .01, *η^2^_p_* = .93). Global orientation was relatively stable for rotations at or near alignment, then fell more steeply to chance with 45º rotation (i.e., no bias to either cardinal orientation). Follow-up analysis indicated that performance at 0º, 12º, and 23º rotation did not differ significantly, whereas performance at 34º and 45º rotation differed significantly from other rotations (HSD = 13.7, *p* < .01).

For arrays constructed from a single-element set, increased element rotation produced a progressive shift in global orientation. Rotation of elements to 23º had little effect on global orientation, where the array was perceived as the mean orientation of elements. Beyond 23º, global orientation became increasingly less stable, where orientation selection declined to chance.

## Experiment 2: Two Element Sets: Proportion of Aligned Elements

The aim of Experiment 2 was to determine whether aligning a proportion of one set affected global orientation similarly across the rotation level of the second set. With a fixed proportion of aligned elements, their input signal to the global structure is constant. If the aligned elements contribute independently to global orientation, the effects of alignment should not vary with the rotation of the second set. For Experiment 2, stimuli contained two element sets, where the proportion of each set varied across conditions.

### Method

Participants. The same five subjects who participated in Experiment 1 also participated in Experiment 2.

Stimuli. The two element sets are labeled here as Set 1 and Set 2. These descriptors were selected in order not to suggest the assignment of a primary and secondary set, or to indicate a target set within a field of randomly-oriented noise elements. Instead, Set 1 and Set 2 both contained elements with an explicit orientation and proportion.

Set 1. Set 1 elements were aligned to the base orientation. Five proportions of Set 1 were tested: 100% (i.e., the entire grid was composed of aligned elements), 80%, 60%, 40%, and 20%.

**Figure 3:**
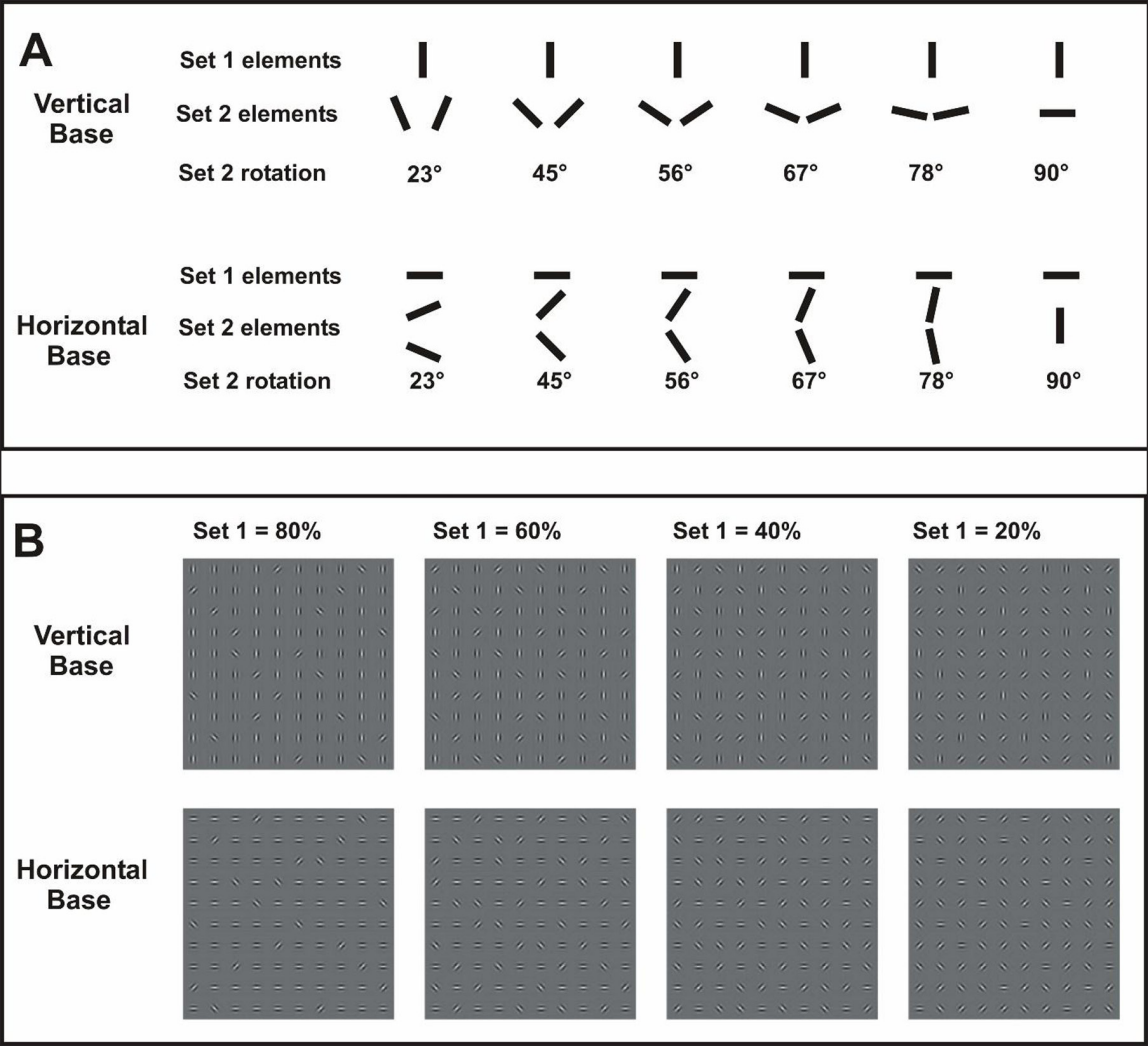
**A**. Element types for Experiment 2 at each level of Set 2 rotation. **B**. Examples of stimuli at each proportion of Set 1. In each case shown here, Set 1 elements are aligned to base orientation, and Set 2 element are rotated 45º.

Set 2. For each Set 1 proportion (with the exception of 100%), Set 2 elements were rotated from the base orientation by 23º, 45º, 56º, 67º, 78º, or 90º, selected randomly on each trial. Figure 3 depicts examples across levels of element set proportion.

Procedure. One of four versions of a stimulus condition was selected randomly without replacement in a block of trials. Performance with each stimulus condition was based upon 24 trials so that there was a total of 600 trials and the duration of the Experiment 2 was about 35 minutes.

### Results and Discussion

**Figure 4:**
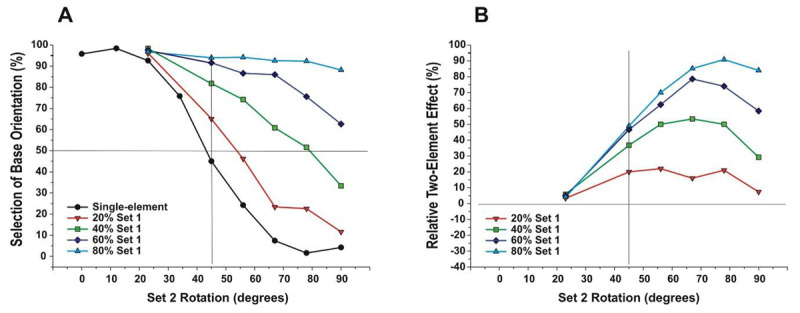
Effects of introducing aligned element among the field of rotated elements. Each Set 1 proportion is indicated with color and shape. **A**. Selection of base orientation as a function of Set 2 rotation. Black circles depict results for single-element condition. **B**. Relative effect of two-element set (two-element set minus one-element set).

Performance for each proportion of aligned Set 1 elements is depicted in Figure 4. In order to examine the effects of two-element sets relative to single-element sets, data from Experiment 1 (single-element set) are included. For the single-element data, in order to depict data for rotations greater than 45°, performance is shown relative to the orthogonal orientation. Results indicate that embedding a proportion of aligned elements among rotated elements stabilized global orientation (i.e., drew global orientation towards the base orientation), and the stabilizing effect progressively increased with the increased proportion of aligned elements (Figure 4A). ANOVA, with repeated measures on the rotation and proportion factors, indicated a main effect of Set 2 rotation (*F*(5,20) = 46.44, *p* < .001, *η^2^_p_* = .92) and a main effect of proportion (*F*(3.12) = 75.35, *p* < .001, *η^2^_p_* = .95). In addition, a significant interaction occurred between rotation and proportion (*F*(15,60) = 10.11, *p* < .001, *η^2^_p_* = .72), where the stabilizing effect of aligned elements increased with increased proportion. Specifically, with 20% Set 1, global orientation differed little from the single-element condition. Selection of the base orientation increased with 40% and 60% Set 1, most notably with Set 2 rotations greater than 45°. With 80% Set 1, Set 2 elements had little effect on global orientation, even when Set 2 elements were orthogonal to the base orientation.

The effects of two-element sets relative to the single-element set from Experiment 1 are depicted in Figure 4B, which illustrate patterns of change in the two-element sets across levels of Set 2 rotation. Deviation from the single-element condition (Figure 4A solid line) represents the effect of introducing aligned elements. Alignment effects were indexed as the performance with a proportion of aligned elements minus performance with all elements rotated (Figure 4B). Results indicate that the alignment effect progressively rose to a peak, then declined with further Set 2 rotation. ANOVA indicated a main effect of Set 2 rotation (*F*(5,20) = 44.71, *p* < .001, *η^2^_p_* = .92) and a main effect of proportion (*F*(3,12) = 62.18, *p* < .001, *η^2^_p_* = .94). A significant interaction also occurred between rotation and proportion (*F*(15,60) = 82.87, *p* < .001, *η^2^_p_* = .95), where increased Set 1 proportion produced a steeper rise in the alignment effect, which peaked at greater levels of Set 2 rotation.

As expected, embedding a proportion of aligned elements stabilized global orientation. However, the stabilizing effect varied with Set 2 orientation. Specifically, the effects of aligned elements (Set 1) rose to a peak with increased Set 2 rotation. The rise in Set 1 effects was steeper, and the peak higher, with a greater proportion of Set 1. Following the peak, Set 1 effects declined, and the point of decline differed with Set 1 proportion. These results indicate that the relative contribution of aligned elements to global orientation varied with Set 2 rotation, indicating an interaction between element sets. With less rotation of Set 2, element sets appear to integrate, and global orientation reflects the mean element orientation across the array. With the increased rotation of Set 2, elements appear to form distinct sets, and global orientation reflects a tradeoff between competing sets. In addition, interactive effects varied across the proportion of Set 1, where element sets appear to integrate with a lower proportion of Set 1.

## Experiment 3: Two Element Sets: Independent Rotation of each Set

The aim of Experiment 3 was to determine whether a deviation from the alignment of one set reduced their contribution to global orientation similarly as that found with the single-element set. If sets operate independently, the effects of alignment should decline as these elements are rotated, regardless of the level of rotation of the second set. Alternatively, if the two sets do not operate independently, but instead interact, then the effects of alignment or deviation from alignment by one set should vary with the rotation of the second set.

### Method

Participants. Five subjects, different from those who participated in Experiments 1 and 2, participated in Experiment 3 (mean age 21 years; age range 19–25 years; 4 females).

Stimuli. Experiment 3 served to examine the effects of independently rotating each element set. Separate measurements were made with 60% and 80% proportion of Set 1.

**Figure 5:**
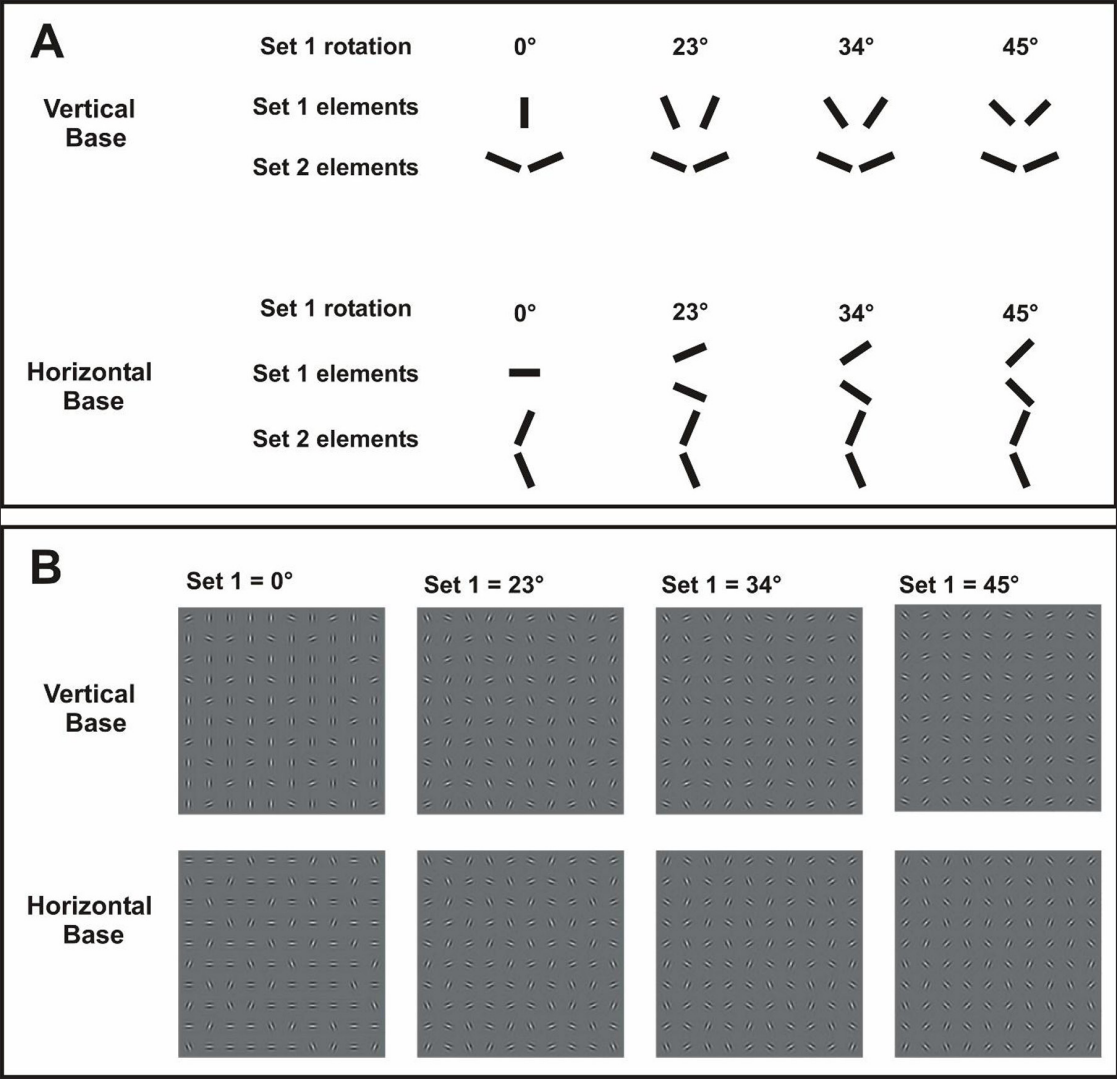
Shown here is an example where Set 2 rotation is 67º, whereas measurements were made with Set 2 rotation set to 0º, 23º, 34º, 45º, 56º, and 67º. **A**. Element types for Experiment 3 at each level of Set 1 rotation. **B**. Examples of stimuli. In each case, Set 1 proportion is 60%.

Set 1. Set 1 elements were rotated by 0º, 23º, 34º, or 45º (Figure 5).

Set 2. For each Set 1 proportion and rotation, Set 2 elements were rotated by 0º, 23º, 34º, 45º, 56º, and 67º.

Procedure. One of four versions of each stimulus condition was selected randomly without replacement in a block of trials for a total of 960 trials. Because of the large number of conditions, each condition was presented on 20 trials. The total duration of the experiment was approximately 45 minutes.

### Results and Discussion

Independent rotation of each set was examined in Experiment 3. Analysis served to determine whether a deviation from the alignment of Set 1 affected global orientation similar to that found with the single-element condition. As such, 23° rotation is expected to produce a little effect, 34° rotation is expected to shift performance towards the single-element function, and 45° rotation should remove the contribution of Set 1, and performance is expected to follow the single-element function. Instead, results showed that 34° and 45° rotation of Set 1 disrupted perceived global orientation. Performance was similar for 0° and 23° rotation of Set 1, although differed with the greater rotation of Set 2.

**Figure 6:**
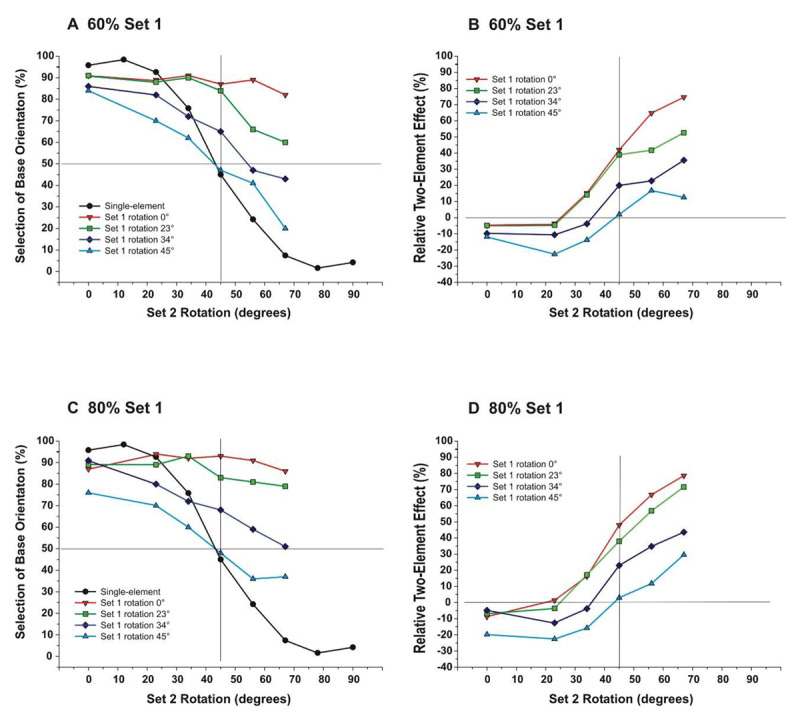
Performance for each level of Set 1 rotation. Each Set 2 rotation is indicated with color and shape. **A** and **C**: Selection of base orientation as a function of Set 2 rotation. Black circles depict results for the single-element condition. **B** and **D**: Relative effect of two-element set (two-element set minus one-element set). Top graphs depict results with 60% Set 1 proportion; bottom row with 80% Set 1 proportion.

Results were similar for 60% (Figures 6A) and 80% (Figure 6C) proportions of Set 1. Included in figures are results of the single-element condition (solid black line). ANOVA, with repeated measures on the rotation and proportion factors, indicated a significant main effect of Set 1 rotation (60% proportion: *F*(3,12) = 64.22, *p* < .001, *η^2^_p_* = .94; 80% proportion: *F*(3,12) = 51.50, *p* < .001, *η^2^_p_* = .93), and a main effect of Set 2 rotation (60% Set 1: *F*(5,20) = 56.97, *p* < .001, *η^2^_p_* = .93; 80% Set 1: *F*(5,20) = 13.44, *p* < .001, *η^2^_p_* = .77). A significant interaction also existed between Set 1 and Set 2 rotation (60% proportion: *F*(15,60) = 4.76, *p* < .001, *η^2^_p_* = .53; 80% proportion: *F*(15,60) = 3.86, *p* < .001, *η^2^_p_* = .49), where rotation of Set 1 compressed the psychometric function. Effects of two element sets varied with the relative rotation of the sets. Specifically, when Set 1 rotation was greater than Set 2, performance was drawn away from the base orientation, where data points fell below the single-element function (Figure 6A and 6C). When Set 1 rotation was less than Set 2, performance was drawn towards the base orientation, where data points fell above the single-element function. The transition point between these effects occurred when Set 1 and 2 rotations were equal, where the two-element functions intersected with the single-element function. The net results were that independent rotation of sets compressed the range of perceived global orientation, pivoting on a transition point along the function describing the single-set condition.

Effects of Set 1 rotation, relative to the single-set condition, varied across levels of Set 2 rotation. Specifically, with increased rotation of Set 1, a greater difference occurred between the single-element and two-element conditions. In addition, this difference was more pronounced with greater rotation of Set 2 (Figure 6B and 6D). ANOVA indicated a significant main effect of Set 1 rotation (60% proportion: *F*(3,12) = 63.81, *p* < .001, *η^2^_p_* = .94; 80% proportion: *F*(3,12) = 52.61, *p* < .001, *η^2^_p_* = .93) and a significant main effect of Set 2 rotation (60% proportion: *F*(5,20) = 162.90, *p* < .001, *η^2^_p_* = .98; 80% proportion: *F*(5,20) = 130.20, *p* < .001, *η^2^_p_* = .97). In addition, a significant interaction existed between Set 1 and Set 2 rotation (60% proportion: *F*(15,60) = 4.81, *p* < .001, *η^2^_p_* = .55; 80% proportion: *F*(15,60) = 3.64, *p* < .001, *η^2^_p_* = .48), where slopes decreased with Set 1 rotation. A decline in Set 1 effects following a peak was not observed, although it is uncertain if this relationship would have been apparent with greater Set 2 rotation.

Results of Experiment 3 indicate that as Set 1 elements deviate from alignment, their contribution to global orientation declined. However, the pattern of change differed from that of rotating the single-element set. Specifically, rotating Set 1 compressed the psychometric function found with the single-element set. These results again indicate an interaction between element sets, which appeared related to their relative rotation. With Set 1 rotated more than Set 2, perceived orientation moved away from the base orientation, whereas the opposite occurred with Set 1 was rotated less than Set 2. As such, with Set 1 rotation, global orientation was more ambiguous.

## General Discussion

The current study served to examine interactions between multiple element sets in the perception of global orientation. Global orientation was based upon an array of two interspersed element sets. Element sets varied in proportion, and were either aligned or rotated relative to a base orientation. Stimuli did not contain noise, but instead both element sets were presented at explicit orientations. Subjects indicated whether the array was oriented predominantly as vertical or horizontal. Three conditions were examined, in which either all elements were rotated (Experiment 1), a proportion of elements remained aligned while remaining elements were rotated (Experiment 2), or each element set was rotated independently (Experiment 3).

Results from Experiment 1 indicated that for the single-element set, global orientation was stable with rotation up to 23º, beyond which responses shifted away from the base orientation. This pattern of change to global orientation serves as a reference by which to examine the pattern of change with the two-element set conditions.

The main findings from Experiment 2 were (1) embedding a set of aligned elements among rotated elements stabilized global orientation, (2) increasing the proportion of aligned elements systematically enhanced the stabilizing effect, and (3) the level of difference between the two-element and single-element sets varied across element rotation, where the difference increased to a peak and then declined. The pattern of difference between the two-element and single element sets suggests changes in the contribution of element sets to global orientation. With minimal rotation, all elements were similar to the base orientation, and the two-element and single-element conditions were similar. With less rotation, less difference existed between aligned and rotated elements, and element sets appear to integrate as a single set (Keeble et al., 1997). With intermediate levels of rotation, aligned elements more strongly contributed to global orientation, causing a greater difference between the two-element and single-element conditions. With the highest levels of rotation, the contribution of aligned elements decreased, and global orientation was weighted more towards rotated elements. These results may reflect an increased saliency of rotated elements as they approached alignment (Harrison & Keeble, 2008).

The main findings from Experiment 3 were (1) increased rotation of Set 1 decreased their stabilizing effect, (2) increased rotation of Set 1 disrupted global orientation for low levels as well as high levels of Set 2 rotation, and (3) differences between the two-element and single-element sets increase across levels of rotation, although these difference were reduced with the rotation of Set 1. This pattern of results occurred when the two-element sets were of similar proportion (60% Set 1, 40% Set 2), as well as when Set 1 dominated the array (80% Set 1, 20% Set 2). Results from Experiment 3 further suggest changes in the relative contribution of element sets as each set was rotated independently. Set 1 provided a greater contribution to global orientation when more closely aligned to the base orientation, whereas its effects decreased with rotation. As with Experiment 2, with the highest levels of Set 2 rotation, the contribution of Set 1 declined.

In all cases, global orientation was not altered for rotations up to 23°. This effect was found for rotation of the single-element set, rotation of Set 2 with aligned Set 1, and rotation of both sets. At similar levels of rotation (up to 18°), multiple element sets appear integrated as a unimodal orientation set (Keeble et al., 1997). Similarly, rotation of 22° approximates the maximum path angle to detect contour segments (Hess & Dakin, 1999; Hess & Field, 1999) as well as the minimum difference in orientation needed to detect texture segmentation, below which texture edges cannot be discriminated (Wolfson & Landy, 1995). Stability in global orientation with minimal element rotation found here is consistent with models of integration by similarly tuned orientation filters (Husk et al., 2012).

Each of the two-element sets forms a grouped pattern that is represented as an integrated structure, such as an association field described for contour integration (Field et al., 1993) or for a response to the sequential presentation of aligned Gabor patches (Chavane et al, 2000). Interactions between sets found here suggest that representations of each set also interact, such that representations of the grouped patterns are modified when presented together.

Interactions among adjacent elements are based upon shared stimulus properties (Adini, Sagi, & Tsodyks, 1997; Polat & Sagi, 1993) that bind common elements to form contours and groups (Angelucci et al., 2002; Hess & Field, 1999, Yao & Li, 2002). Deviation from alignment reduces connectivity, which weakens interactions among adjacent elements (Polat & Sagi, 1993). For stimuli used here, global orientation was based upon element integration across a relatively broad stimulus field. With interspersed element sets, discontinuities in orientation exist among adjacent elements, where strings of common elements are interrupted by members of the other element set. Such discontinuities reduce local integration, and global orientation is based upon more wide-spread relationships across the stimulus array. The degree of difference between element sets appears to guide whether sets integrate as a new unified structure, or whether multiple representations compete (Rashal et al., 2017). With a similar orientation of element sets, global patterns are based upon integration across all elements. With contrasting element sets, distinct integrated fields, whose elements are broadly distributed and interspersed, compete in the perception of global orientation.

## Figures and Tables

**Table 1:** Summary of stimulus parameters for each experiment.

	Set 1 Proportion (%)	Set 1 Rotation (°)	Set 2 Proportion (%)	Set 2 Rotation (°)

Experiment 1	100	0	–	–
	100	12	–	–
	100	23	–	–
	100	34	–	–
	100	45	–	–
Experiment 2	100	0	–	–
	80	0	20	23, 45, 56, 67, 78, 90
	60	0	40	23, 45, 56, 67, 78, 90
	40	0	60	23, 45, 56, 67, 78, 90
	20	0	80	23, 45, 56, 67, 78, 90
Experiment 3	80	0	20	0, 23, 34, 45, 56, 67
	80	23	20	0, 23, 34, 45, 56, 67
	80	34	20	0, 23, 34, 45, 56, 67
	80	45	20	0, 23, 34, 45, 56, 67
	60	0	40	0, 23, 34, 45, 56, 67
	60	23	40	0, 23, 34, 45, 56, 67
	60	34	40	0, 23, 34, 45, 56, 67
	60	45	40	0, 23, 34, 45, 56, 67

## Data Availability

Raw data of each experiment are uploaded to Open Science Framework with a unique identifier 2dp6e (https://osf.io/2dp6e/) which is publicly available.
